# Arrhythmia ECG Noise Reduction by Ensemble Empirical Mode Decomposition

**DOI:** 10.3390/s100606063

**Published:** 2010-06-17

**Authors:** Kang-Ming Chang

**Affiliations:** Department of Photonics and Communication Engineering, Asia University, Wufeng, Taichung County, 500, Lioufeng Rd., Wufeng, Taichung County, 41354, Taiwan

**Keywords:** arrhythmia ECG, ensemble empirical mode decomposition, composite noise, filter

## Abstract

A novel noise filtering algorithm based on ensemble empirical mode decomposition (EEMD) is proposed to remove artifacts in electrocardiogram (ECG) traces. Three noise patterns with different power—50 Hz, EMG, and base line wander – were embedded into simulated and real ECG signals. Traditional IIR filter, Wiener filter, empirical mode decomposition (EMD) and EEMD were used to compare filtering performance. Mean square error between clean and filtered ECGs was used as filtering performance indexes. Results showed that high noise reduction is the major advantage of the EEMD based filter, especially on arrhythmia ECGs.

## 1. Introduction

Empirical mode decomposition (EMD) is a novel recently developed algorithm [1]. EMD is based on a decomposition derived from the data and is useful for the analysis of nonlinear and nonstationary time series signals [2]. With iterative decomposition of signals, EMD separates the full signal into ordered elements with frequencies ranged from higher to lower frequencies in each intrinsic mode function (IMF) level. Different from the classical Fourier decomposition with sine and cosine basis functions, EMD depends on the characteristics of the signal; therefore EMD behaves as a filter bank without a predefined cut-off frequency [2]. This interesting property of EMD has been widely applied in biomedical signal analysis, such as monitoring the effect of anesthetic drugs [3], rapid screening of obstructive sleep apnea [4], and respiratory sinus arrhythmia estimation from ECGs [5].

EMD is also used for ECG noise reduction [6–9]. Blanco-Velasco developed an EMD-based algorithm to remove the baseline wander and high-frequency noise of ECGs [10]. Nimunkar and Tompkin added a pseudo-high-frequency noise to IMFs as an aid to remove power-line noise. They also developed a complete ECG processing algorithm for R-peak detection and feature extraction, based on EMD approaches [11]. Owing to the fact that the lower IMF levels correspond to higher frequency components and *vice versa*, reconstruction without the lower IMF level can remove high-frequency noise. Thus, low-frequency baseline wander can be removed by reconstruction without higher IMF levels [12].

The major disadvantage of EMD is the so-called mode mixing effect. Mode mixing indicates that oscillations of different time scales coexist in a given IMF, or that oscillations with the same time scale have been assigned to different IMFs. Hence, ensemble EMD (EEMD) was introduced to remove the mode-mixing effect [13]. The principle of the EEMD is to add white noise into the signal with many trials. The noise in each trial is different, and the added noise can be canceled out on average, if the number of trials is sufficient. Thus, as more and more trials are added to the ensemble, the residual part is the signal. EEMD was also widely used for signal processing. For example, reconstruction from selected IMFs was used for the evaluation of pipelines utilizing the magnetic flux leakage (MFL) technique [14]. EEMD was also been used to simulate cardio-respiratory signals in order to measure cardiac stroke volume. EEMD improved them better than EMD by mode mixing removal [15].

Arrhythmia ECGs have different ECG patterns than the normal state. Different arrhythmia states, such as premature arrhythmias, superavent arrhythmias, ventricular arrhythmias and conduction arrhythmias, present various ECG waveforms. During the ECG measurement, various types of noises, such as muscle noise, baseline wander, and power-line interferences, are recorded in the ECG signals, interfering with the ECG-information identification. Numerous signal-processing methods have been used on the studies of ECG noise reduction, especially on arrhythmia ECGs. Adaptive regression and the corresponding Kalman recursions were used to remove ventricular fibrillation (VF) electrocardiogram (ECG) signal noise [16]. Multichannel Wiener filter and a matching pursuit-like approach were applied to remove cardiopulmonary resuscitation artifacts from human ECGs [17]. The adaptive LMS filter used to remove cardiopulmonary resuscitation (CPR) artifacts from ECGs has achieved high sensitivity and specificity of around 95% and 85%, respectively [18]. Another adaptive filter based filter to suppress random noise in electrocardiographic (ECG) signals, unbiased and normalized adaptive noise reduction, can effectively eliminate random noise in ambulatory ECG recordings, leading to a higher SNR improvement than possible with a traditional LMS filter [19]. The time-frequency plane was also used to separate signal and noise components with an entire ensemble of repetitive ECG records, based on a Wiener filter. High noise reduction and low signal distortion was achieved after ensemble averaging problem involving repetitive deterministic signals mixed with uncorrelated noise [20].

The goal of this study is to investigate EEMD based filtering performance and the corresponding phase delay of filtered signals in arrhythmia ECGs. Low pass, high pass and band pass filters were designed to meet various noises conditions: muscle contraction, 50 Hz power line and baseline wonder. Traditional Butterworth filter and Wiener filter was also used to compare the filtering performance. Phase distortion of the filtered ECG was also investigated.

## 2. EMD and EEMD algorithm

### 2.1. EMD

The EMD algorithm used in this study comprises the following steps [1]:

1. Identify all the extrema (maxima and minima) of the signal, *x*(*s*).
2. Generate the upper and lower envelope by the cubic spline interpolation of the extrema point developed in step (1).
3. Calculate the mean function of the upper and lower envelope, *m*(*t*).
4. Calculate the difference signal *d*(*t*) = *x*(*t*)−*m*(*t*).
5. If *d*(*t*) becomes a zero-mean process, then the iteration stop and *d*(*t*) is an IMF1, named *c*_1_(*t*); otherwise, go to step (1) and replace *x*(*t*) with *d*(*t*).
6. Calculate the residue signal *r*(*t*) = *x*(*t*)−*c*_1_(*t*).
7. Repeat the procedure from steps (1) to (6) to obtain IMF2, named *c*_2_(*t*). To obtain *c_n_*(*t*), continue steps (1)–(6) after n iterations. The process is stopped when the final residual signal *r*(*t*) is obtained as a monotonic function.

At the end of the procedure, we have a residue *r*(*t*) and a collection of *n* IMF, named from *c*_1_(*t*) to *c_n_*(*t*). Now, the original signal can be represented as:

(1) $$x\left(t\right)=\sum_{i=1}^{n}\;c_{i}(t)+r(t)$$

Often, we can regard *r*(*t*) as *c*_*n*+1_(*t*).

### 2.2. EEMD

According to Wu [9], the steps for the EEMD algorithm are as follows:

1. Add a white noise series *n*(*t*) to the targeted signal, named *x*_1_(*t*) in the following description, and *x*_2_(*t*)=*x*_1_(*t*)+*n*(*t*).
2. Decompose the data *x*_2_(*t*) by EMD algorithm, as described in Section 2.1.
3. Repeat Steps (1) and (2) until the trial numbers, each time with different added white noise series of the same power at each time. The new IMF combination *C_ij_*(*t*) is achieved, where *i* is the iteration number and *j* is the IMF scale.
4. Estimate the mean (ensemble) of the final IMF of the decompositions as the desired output:
  (2) $${\mathrm{EEMD\_c}}_{j}(t)=\sum_{i=1}^{\mathrm{ni}}\;c_{\mathrm{ij}}(t)$$
  where *ni* denotes the trial numbers.

## 3. Method

**Figure 1.**
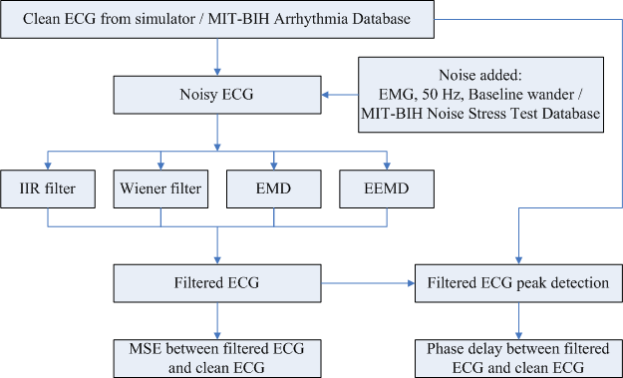
Flowchart of this study.

A simulated arrhythmia ECG segment with designed noises was used to examine filter output. Noises contained EMG, 50 Hz power line and baseline wanders. Low pass filter, high pass filter and band pass filters were designed with Butterworth filter, Wiener filter, EMD and EEMD based filters. The filtering performance was compared. The overall flowchart is shown in Figure 1. The detailed description is given in the following sub-section.

### 3.1. Simulated Arrhythmia ECG and Noise Data

#### A. Clean synthetic ECG signal:

**Figure 2.**
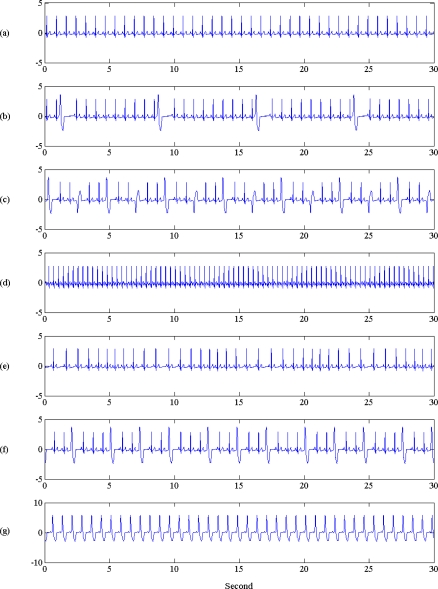
Illustration of normal and arrhythmia ECG signals used in this study. Signal durations are 30 s. From top to bottom: (a) normal ECG, (b) premature arrhythmia with PVC1, denoted as P1, (c) premature arrhythmia with multifocal PVC, denoted as P2, (d) superavent arrhythmia with atrial tach, denoted as S1, (e) superavent arrhythmia with sinus arrhythmia, denoted as S2, (f) ventricular arrhythmia with 24 PVCs per min, denoted as V1, (g) conduction arrhythmia with Lf bundle branch block, denoted as C1.

Simulated normal and arrhythmia ECGs were derived from a ECG simulator (type number BC Biomedical PS-2210 Patient Simulator) with 60 s duration. The ECG simulator parameter was 80 BPM, temperature 37 °C, Maximum peak to minimum peak voltage was 5 mV, breath Rate was set at 30. There are one normal ECG, and six arrhythmia ECGs, such as premature arrhythmias, superavent arrhythmias, ventricular arrhythmias and conduction arrhythmias. The ECG segment is shown in Figure 2. The corresponding ECG label and disease type was also described in the caption of Figure 2.

#### B. Real ECG database

Real ECG data was derived from an arrhythmia ECG database. Number 101, 102 and 103 and 104 were used. A band-pass filter ranged 1–35 Hz was used as preprocessing filter. The cleaned ECG was then used a real ECG template. The signal was 30 min durations. [21].

#### C. Synthetic noises:

High frequency ECG noise types, such as muscle contraction and 50 Hz power line interference, and low frequency ECG, baseline wander were investigated in the following session. All noises were also reduced to three noise levels, 25%, 50% and 100%, with respect to the maximum noise level. The maximum noise level was predetermined as an amplitude ratio with respect to normal ECG, Vpp, which is amplitude of maximum peak to minimum peak. The noise simulation algorithm was similar to the suggestion in [22]:

1. EMG noise: EMG noise was model by a random number with normal distribution, originally manipulated with the Matlab code *randn.m*. The maximum EMG noise level was the scaling of random sequence and the multiplication to Vpp with reduced ratio of 1/8. EMG noise sequence was denoted as N1(t).
2. Power line noise: Power line interference was modeled by 50 Hz sinusoidal function with multiplication on amplitude derived with Matlab code *rand.m*. The maximum 50 Hz noise level was the scaling of random sequence and the multiplication to Vpp with reduced ratio of 1/4. 50 Hz noise sequence was denoted as N2(t).
3. Baseline wander: Baseline wander was model by a Baseline wander a 0.333 Hz sinusoidal function. The maximum noise level was the same amplitude scale with Vpp. Baseline wander was denoted as N3(t).
4. Composite noise: Composite noise was the combination of the above three noise with the following relation:
  (3) N4(t) =0.5*[N1(t)+N2(t)]+N3(t)

**Figure 3.**
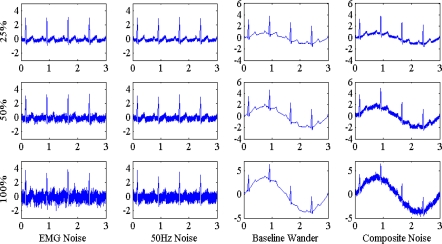
Illustration of EMG noise, 50 Hz noise, baseline wander and composite of three noises on normal ECG. Noise levels of 25%, 50% and 100% are added, respectively. Signal durations are 3 s.

Illustration of the four noises with three levels on a normal ECG is shown in Figure 3.

#### D. Real noise database

Real noises are extracted from the noise stress test database in MIT-BIH [23]. There are three noise patterns: baseline wander (in record “bw”), muscle (EMG) artifact (in record “ma”), and electrode motion artifact (in record “em”). Both one minute and total 30 min duration noises were selected, respectively. The short one minute noise was used for synthetic ECG and the 30 min duration noise was for real ECG signal derived from the arrhythmia ECG database.

### 3.2. EMD/EEMD Based Filtering Algorithm

ECG was filtered (reconstructed) with partial reconstruction IMF by EMD, EEMD respectively with following equation:

(4) $${\mathrm{RECG\_emd}}_{\mathrm{kq}}\;(t)=\sum_{i=k}^{q}\;c_{i}(t),$$

(5) $${\mathrm{RECG\_eemd}}_{\mathrm{kq}}\;(t)=\sum_{i=k}^{q}\;{\mathrm{EEMD\_c}}_{i}\;(t),$$

When *k* = 1, q = n, RECG_emd_1q_ becomes equivalent to the original noised ECG. A low pass filter was derived from deletion of lower IMF scale, than means k > 1; A high pass filter was derived from deletion of high IMF scale, than means q < n; and a band pass filter was consequently with middle part of IMF scales, that means both conditions k > 1 and q < n must be satisfied. The optimal choice of (k,q) pairs for each filter was determined with minimum MSE by sequential search approach. The EEMD parameters was 10 dB added white noise and 200 times trial number, according to previous study [24].

### 3.3. Wiener Filter

The formula of the Wiener filter is given as [25]:

(6) $$w=R_{X_{1}\;X_{1}}^{-1}\;R_{X_{1}\;X}$$

where *w* is the Wiener filter coefficients, and the cross correlation of *x*_1_(*t*) and *x*(*t*), *R*_*X*_1_*X*_, autocorrelation of *x*_1_(*t*), *R*_*X*_1_*X*_1__ were estimated. The *x*_1_(*t*) and *x*(*t*), represent the input signal and desired signal corresponding to *x*_1_(*t*) and *x*(*t*) introduced in the earlier section, respectively. Wiener filter theory is based on the minimization of difference between the filtered output and desired output. Filter coefficient was estimated by the least mean squares method on the square of the difference between the desired and the actual signal after filtering. In this study, the Wiener filter was derived from Matlab function *firwiener.m,* with filter order 300.

### 3.4. Traditional IIR Filter

A Butterworth filter was used with three filter speculation. The low pass filter was a 10th order Butterworth filter with a 35 Hz cutoff frequency, and the high pass filter was a 3rd order Butterworth filter with a 1 Hz cutoff frequency. The band pass filter was the cascade computation result of the low pass filter and high pass filter.

### 3.5. Filtering Performance Index

Three are two indexes used to indicate the filter performance on EEMD and the other filter, one is mean square error (MSE) and the other is phase delay. MSE was to measure the difference between the original “clean” ECG and the reconstructed ECG. MSE is mainly from the residual noise and also ECG distortion after filtering process. MSE can be defined as follows:

(7) $$\mathrm{MSE}=\frac{\sum_{t=0}^{L-1}{\left(x\left(t\right)-\overset{\wedge }{x}\left(t\right)\right)}^{2}}{L}$$

where the nominator part is the square error, and *x̑*(*t*) is the reconstructed ECG, such as *RECG_emd_kq_* or *RECG_eemd_kq_* in Equations (4) and (5). The phase delay of *x̑*(*t*) was also calibrated before MSE calculation. *L* is the length of the signal. The lower the MSE value, the higher filtering performance was evaluated for filters. Another quantitative feature, MSE_QRS_ is also defined as the MSE within the windowed QRS complex:

(8) $${\mathrm{MSE}}_{\mathrm{QRS}}=\frac{\sum_{k=1}^{L}\sum_{t=0}^{W-1}{\left(y\left(t\right)-\overset{\wedge }{y}\left(t\right)\right)}^{2}}{L*W}$$

where L is the number of QRS complex, and W is the window duration of each QRS complex. MSE_QRS_ is used to measure the recovery performance of QRS complex with various filter method.

## 4. Results:

### 4.1. EMD and EEMD Decomposition:

**Figure 4.**
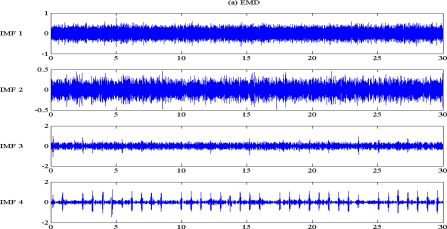

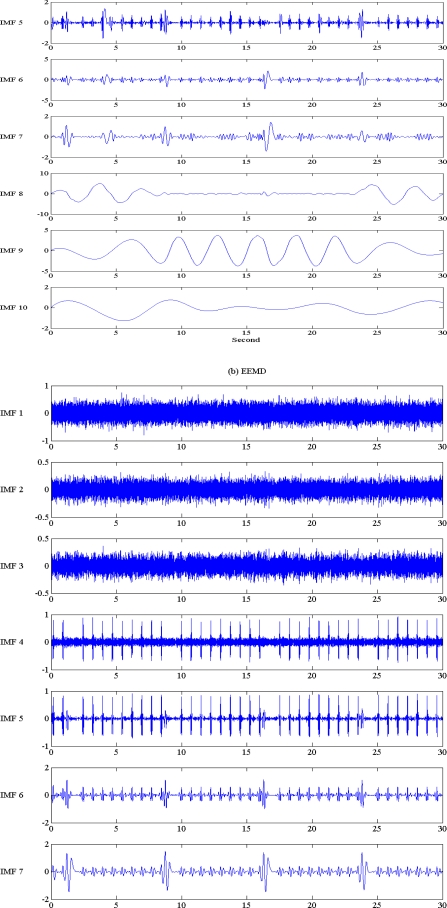

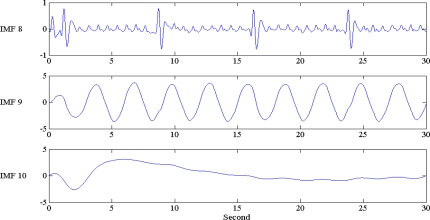
Illustration of IMF distribution of a ventricular arrhythmia ECG (V1) with 100% composite noise by: (a) EMD, and (b) EEMD. From top to bottom is low level IMF to high level IMF. Signal durations are 30 s.

The typical EMD and EEMD decomposition and extracted IMF are illustrated in Figure 4. The low level IMF contained high frequency components; while the high level IMF contained low frequency components. IMF distribution is very similar to a filter bank. Unlike a traditional filter bank, and similar sub-band decomposition algorithms, such as wavelets, IMF is not band restricted. Adaptive decomposition based on the signal pattern complexity is the main feature of IMF. Not specific IMF level would contain pre-determined frequency range components, that means an adaptive frequency range filtering process.

**Figure 5.**
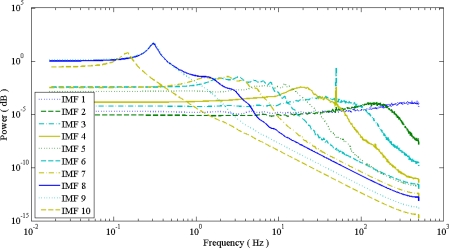

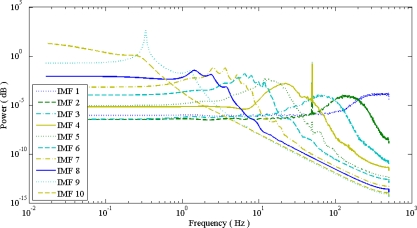
Corresponding IMF spectrum distribution of (a) EMD, and (b) EEMD of Figure 4. The x-axis unit is Hz, y-axis is power. There is less spectrum overlapping of EEMD than EMD due to reduction of mode mixing in EMD.

The difference between EMD and EEMD is the mode mixing reduction of EEMD. Comparing the IMF component of the same level, EEMD has more concentrated and band limited components. High frequency noises are more localized in the low IMF level. That can be seen from Figure 5, the corresponding IMF spectrum distribution of EMD and EEMD. The 50 Hz spike is in the 1st–4th level in EMD and EEMD; while the 0.33 Hz baseline wander is in the 8th and 9th level in EMD and only in 9th level in EEMD. ECG components are located between the 4th to 7th level in EMD and 4th to 8th level in EEMD.

### 4.2. MSE Performance:

**Figure 6.**
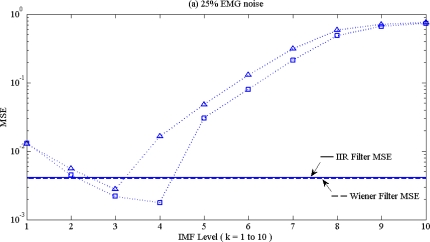

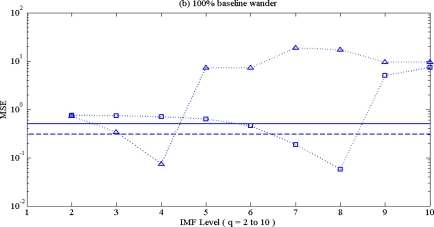
MSE distribution of ventricular arrhythmia ECG (V1) with (a) 25% EMG noise, (b) 100% baseline wander for EMD (dot line with triangle mark), EEMD (dot line with square mark),on different IMF levels. Corresponding MSE of Wiener filter (dash line) and IIR filter (solid line) with low pass filter spec are also shown in (a) and in (b) with high pass filter spec. The minimum MSE of EMG noise is at k = 3 for EMD and k = 4 for EEMD, and minimum MSE of baseline wander is at q = 8 (EEMD) and q = 4 (EMD), respectively.

Noise reduction performance was evaluated by MSE. MSE performance of low pass filter and high pass filter spec is represented in Figure 6(a) and Figure 6(b). As shown in Figure 6(a), as IMF level increased, MSE value would be decreased due to the remove of high frequency noise components; while k increased and signal components were also be deleted, MSE increased due to signal distortion. Therefore the optimal IMF level was chosen on the concave with minimum MSE value, and an EMD/EEMD based low pass filter was determined. For the same reason, a high pass filter was also determined with another concave with the deletion from high IM level.

**Figure 7.**
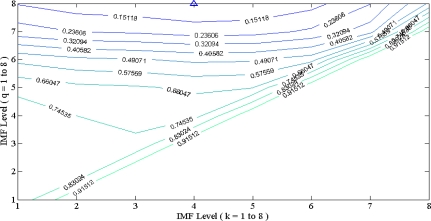
Contour map of MSE under various (k,q) pairs for Ventricular arrhythmia ECG (V1) with 100% composite noise by EEMD. The (k,q) location with lowest MSE was triangle mark. In this case, k = 4 and q = 8 was the optimal solution.

EMD and EEMD based band pass filter need the reduction of both high level and low level IMF, and optimally the middle part of IMF would be conserved, that corresponds to the clean ECG component. Sequential search of MSE with all possible (k,q) combinations was evaluated. A contour map with x-axis as k and y-axis as q was sketched. A minimum MSE point to indicated the optimal (k,q) pair location is achieved for the optimal band pass filtering performance and is illustrated in Figure 7.

**Figure 8.**
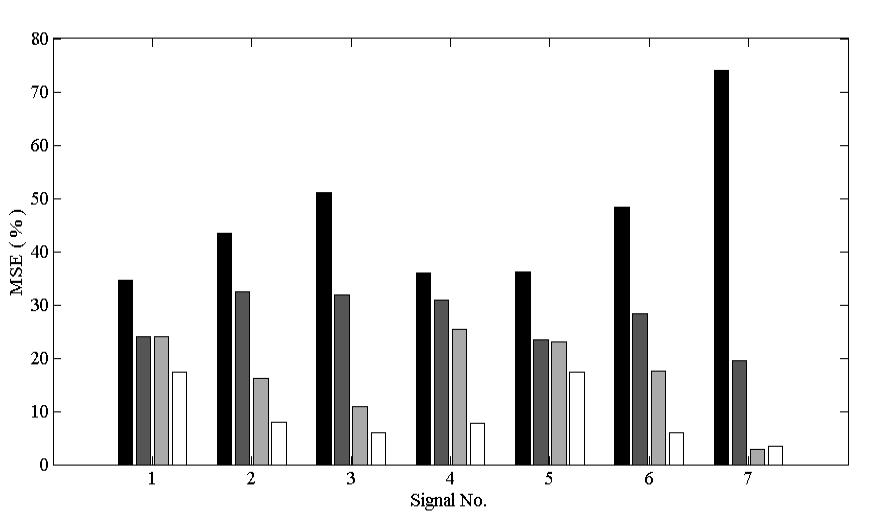
MSE percentage of all ECG contaminated with 100% composite noise. X-axis is the seven ECG segments, the same sequence with that shown in Figure 2. Each ECG segment has average MSE of four filters, from left to right sites are IIR, Wiener, EMD and EEMD. EEMD always has lowest MSE percentage among the four filters, and it is always true for normal ECG, and also for arrhythmia ECG.

Figure 6 shows that the MSE ranking from high to low was IIR > Wiener > EMD > EEMD. EEMD always has lowest MSE under various noise contamination scenarios, which perform as low pass filter, high pass filter and also as band pass filter. This result, indicating that EEMD is also superior to other filters, not only for normal ECG, but also performs well for arrhythmia ECG, is also shown in Figure 8. That means EEMD has good noise reduction performance, under various ECG patterns. With the deletion of low IMF level, EEMD performed as a low pass filter; while with the deletion of high IMF level, EEMD performed as high pass filter. Sequential search of lowest MSE on (k,q) pairs also indicated the optimal band pass filter performance.

The detailed MSE values for synthetic ECG are listed in Table 1. From the results of Table 1, EEMD performed better with light noise percentage, and also better on baseline wander than on high frequency noise, both on 50 Hz interference and EMG noise.

**Figure 9.**
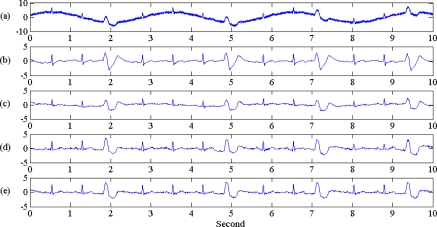
From top to bottom: (a) ventricular arrhythmia ECG (V1) with 100% composite noise and corresponding filter output by (b) IIR, (c) Wiener, (d) EMD (k = 4, q = 7) and (e) EEMD (k = 4, q = 8).

For real noises, baseline wander, muscle contraction and motion artifact, EEMD still had lowest MSE performance than other filters with synthetic V1 signal. The typical filtered ECG of the four filters used in this study is sketched in Figure 9. The IIR filtered ECG has some waveform distortion, especially on the S peak, and there is a pseudo positive peak on PVC pattern; while the output of EMD and EEMD remaining similar to the original ECG signal pattern. That is the advantage of EMD and EEMD with the near zero phase delay character.

**Figure 10.**
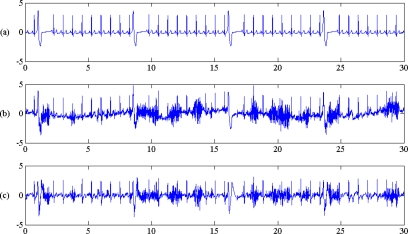

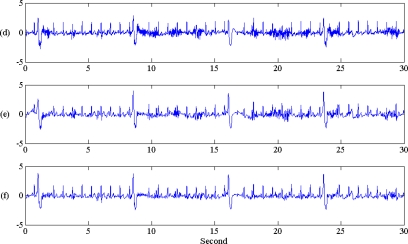
From top to bottom are (a) ventricular arrhythmia ECG (V1), (b) with muscle artifact ’ma’ noise and corresponding filter output by (c) IIR, (d) Wiener, (e) EMD and (f) EEMD.

The filter output for V1 with real noise corruption is shown in Figure 10. It is obvious that EEMD has better filtering performance under muscle contraction contamination.

The QRS complex recovery for synthetic ECG is organized in Table 2. It is obvious from the data in this Table that MSE is higher with higher noise percentage. For baseline noise and composite noise, EEMD has lowest MSE than the other approach. There is no significant difference between the four filter methods on MSE_QRS_ result for real noise contamination. The MSE performance of real noises on the real ECG database is shown in Table 3. Like Table 1, EEMD still has the lowest MSE than the other filters.

## 5. Discussion:

This article investigated the effect of EEMD filtering both on normal ECG and arrhythmia ECG. In additional to normal ECG, EEMD seem more useful on arrhythmia ECG filtering. Arrhythmia ECG with composite noise is the most common case during clinical ECG measurement. Not only is the lower MSE performance, but also on conservation of filtered ECG waveform performed by EEMD. In this study, signal P1, P2, V1, C1 displayed impressive filtering advantages with EEMD, especially on the PVC peak. There are some pseudo peaks produced by the IIR filter, especially on the S peak and a pseudo PVC positive peak. These pseudo peaks could lead to an improper medical diagnosis.

Mode mixing reduction between adjacent IMF levels is the main advantage of EEMD over traditional filters on arrhythmia ECG noise filtering. With the higher computation effort, it leads to better filtering performance. Due to the added noise used during EEMD, there is better filtering performance for EEMD on low noise power conditions.

ECG noise reduction procedure by EEMD on arrhythmia ECG with composite noise was proposed in this article. Something similar has been proposed based on EMD [10], but EEMD had better filtering performance than EMD by reducing mode mixing. The previous study was devoted to the high frequency noises, this study has tried to include the baseline wander noise and extend the signal to arrhythmia ECG. The criterion to achieve an optimal EEMD level selection rule is also proposed. For low pass filtering, iterative deletion of low level IMF until a minimum MSE is reached. The same method is used for high pass filtering, but with deletion of high level IMF to reserve the high frequency component in the low IMF level. It is a little time-consuming to obtain the optimal band pass filtering criteria on suitable IMF levels, but it can be replaced by visual inspection with relative IMF components. From Figure 4 it can be seen that level 4 to level 7 on the EMD contain R peak information, seen in level 4 to level 8 in EEMD. Therefore a smart guess of (k,q) pairs with slight IMF level modification may be necessary to achieve the minimum MSE points with less computation. In the future, optimal selection criteria of IMF level in an interesting issue. Since each IMF is a filter-like output, it is reasonable to expect a predictable IMF level for ECG noise reduction. Unfortunately, the frequency range of each IMF level is not “predictable”, unlike traditional filter banks; therefore there is no criterion now to predict an optimal IMF level for noise reduction. This will be a challenging topic to be investigated in future work.

## 6. Conclusions

This paper proposes a high performance and easy implemented ECG noise reduction procedure based on EEMD. Application of EEMD with adaptive IMF basis properties also has potential for other biomedical signals or other fields. For arrhythmia ECG with PVC it is more useful to use EEMD to remove composite noise than traditional filters. Although EEMD has a heavy computational load, it is still suitable for getting better noise reduction performance on arrhythmia ECG under off line analysis.

## Figures and Tables

**Table 1.** MSE result of Ventricular arrhythmia ECG of four filter methods with four noises. Minimum MSE of the same noise are mark bold.

**Noise type**	**Noise percentage**	**IIR**	**Wiener**	**EMD (IMF level)**	**EEMD (IMF level)**

EMG (* E-3)	25 %	4.1	4.0	2.8 (k = 3)	**1.8** (k = 4)
	50%	6.6	12.3	11.1 (k = 4)	**4.6** (k = 4)
	100%	18.1	34.4	24.6 (k = 4)	**18.1** (k = 4)

50 Hz (* E-3)	25 %	3.3	**1.0**	7.2 (k = 2)	2.0 (k = 4)
	50%	3.8	3.0	11.9 (k = 4)	**3.0** (k = 4)
	100%	5.7	9.4	10.7 (k = 4)	**5.1** (k = 4)

Baseline (* E-2)	25 %	49.5	10.1	3.0 (q = 5)	**2.3** (q = 9)
	50%	49.7	18.4	8.5 (q = 5)	**4.8** (q = 8)
	100%	50.5	30.4	7.4 (q = 4)	**5.7** (q = 8)

Composite (* E-2)	25 %	52.6	10.3	8.5 (k = 3, q = 8)	**2.3** (k = 4, q = 9)
	50%	52.8	18.8	5.5 (k = 3, q = 8)	**5.0** (k = 4, q = 8)
	100%	54.2	31.7	19.5 (k = 4, q = 7)	**6.6** (k = 4, q = 8)

“em”(* E-2)	100%	49.1	19.6	19.3 (k = 1, q = 5)	**16.3** (k = 4, q = 7)
“ma” (* E-2)	100%	36.1	10.7	13.3 (k = 3, q = 7)	**8.5** (k = 5, q = 9)
“bw” (* E-2)	100 %	23.1	6.9	2.7 (k = 1, q = 7)	**1.5** (k = 4, q = 9)

**Table 2.** MSE_QRS_ result of ventricular arrhythmia ECG of four filter methods with synthetic 100% composite noise and real noises. Minimum MSE of the same noise are mark bold.

Noise type	Noise percentage	IIR	Wiener	EMD	EEMD
EMG (* E-3)	25 %	**9.5**	19.1	19.7	18.6
	50%	**12.0**	65.1	75.8	67.7
	100%	**44.6**	140.5	172.3	164.4
50Hz (* E-3)	25 %	8.6	**2.1**	8.4	3.4
	50%	**9.1**	26.2	41.4	27.8
	100%	12.2	**11.9**	19.2	12.0
Baseline (* E-2)	25 %	53.4	9.3	2.4	**1.6**
	50%	53.7	19.4	7.0	**4.5**
	100%	54.5	34.5	7.5	**6.1**
Composite (* E-2)	25 %	57.7	9.5	5.7	**1.6**
	50%	58.2	20.3	5.1	**4.8**
	100%	58.9	37.7	18.6	**7.1**
‘em’(* E-2)	100%	63.6	**33.4**	38.7	34.5
‘ma’ (* E-2)	100%	54.9	**41.4**	55.7	50.8
‘bw’ (* E-2)	100 %	**22.9**	34.7	42.9	41.7

**Table 3.** MSE result of real ECG of four filter methods with three real noises. Minimum MSE of the same noise are mark bold.

Signal	Noise	IIR	Wiener	EMD	EEMD
101 (* E-2)	em	14.7	6.7	8.6	**6.5**
	ma	6.0	2.4	3.6	**2.2**
	bw	4.1	1.6	1.0	**0.7**
102 (* E-2)	em	18.2	**7.5**	10.9	7.9
	ma	9.5	2.3	3.2	**1.9**
	bw	7.6	1.9	1.3	**0.8**
103 (* E-2)	em	13.7	6.2	7.3	**5.7**
	ma	5.0	**2.4**	3.7	2.8
	bw	3.1	1.3	1.0	**0.6**
